# Transcendental Meditation for the improvement of health and wellbeing in community-dwelling dementia caregivers [TRANSCENDENT]: a randomised wait-list controlled trial

**DOI:** 10.1186/s12906-015-0666-8

**Published:** 2015-05-08

**Authors:** Matthew J. Leach, Andrew Francis, Tahereh Ziaian

**Affiliations:** School of Nursing & Midwifery, University of South Australia, Adelaide, Australia; School of Health Sciences, RMIT University, Bundoora, VIC Australia

**Keywords:** Affect, Caregiver, Carer, Clinical trial, Cognitive performance, Dementia, Quality of life, Stress, Randomised controlled trial, Transcendental meditation

## Abstract

**Background:**

Dementia is a prevalent neurodegenerative disorder affecting an estimated 24.3 million people across the globe. The burden on those caring for people with dementia is substantial, with widespread implications for the caregiver, the care recipient and the community. Relaxation techniques, such as Transcendental Meditation® (TM), have been shown to reduce stress and anxiety in healthy workers; similar benefits are anticipated in dementia caregivers. The objective of this study was to ascertain whether TM can improve psychological stress, quality of life, affect and cognitive performance in dementia caregivers.

**Methods:**

The study was conducted as a pilot prospective, multi-centre, community-based, randomised wait-list controlled trial. Community-dwelling caregivers of persons with diagnosed dementia were randomly assigned to a 12-week (14-hour) TM training program or wait-list control. Participants were assessed for quality of life, stress, affect, cognitive performance and adverse effects. The feasibility of the study was also evaluated.

**Results:**

Seventeen caregivers were recruited and randomised. Improvements in WebNeuro response speed scores over time were significantly (*p* = 0.03) greater in the TM group relative to control. Changes between groups over time in all other primary and secondary outcome measures did not reach statistical significance. However, there was a trend toward greater improvement in WebNeuro stress, depression and negativity bias scores in the TM group. Adverse events were reported amongst 63 % of TM-treated subjects; however, events were generally transient, of mild-moderate intensity and only ‘possibly’ related to TM.

**Conclusions:**

Dementia caregivers exposed to TM demonstrated varying degrees of improvement in several measures of cognitive function, mood, quality of life and stress following exposure to TM. However, as the pilot study was underpowered, no firm conclusions can be made about the effectiveness of TM in this caregiver population. Findings from full-scale trials are now warranted.

**Trial registration:**

Australian New Zealand Clinical Trial Registry ACTRN12613000184774 (Registered 15th February 2013).

## Background

Dementia (recently re-termed neurocognitive disorder) [1] is a syndrome characterised by progressive decline in global cognitive ability and increasing dependence on others (such as family) for completion of instrumental activities of daily living. It is estimated that more than 24.3 million people across the globe suffer from the disorder; predicted to rise to over 81.1 million by 2040 [2]. In Australia, 266,000 people have dementia [3], which accounts for 5.3 % of all disability-adjusted life years lost due to disease in older Australians [4]. The combination of chronicity and prevalence contributes to a substantial disease burden, with the total financial cost of dementia care in Australia in 2011 predicted to be close to AU$10.8 billion (US$11.5 billion).

The disease burden for relatives of dementia sufferers can be significant, with associated stress in particular being consistently identified as a key source of financial hardship in family caregivers (i.e. from reduced working hours or relinquished employment) [5], as well as physical and psychological morbidity, including sleep disturbance, depression, anxiety, social isolation, comorbid illness, impaired cognitive performance, and reduced quality of life [6–9]. Whilst stress-management programs for this group are prevalent, they often involve substantial ongoing time-investment and re-training from time-poor individuals; consequently, such programs are subject to poor retention rates and modest overall effectiveness [10].

A sustainable alternative to stress-management in this target group, which is potentially low-cost, relatively effortless, easily implemented and convenient (i.e. can be practiced anywhere and anytime), is Transcendental Meditation® (TM) [11]. This highly-standardised, automatic self-transcending form of meditation allows an individual’s attention to drift to a less active, quieter style of mental functioning, to yield a unique psychophysiological state of restful alertness [11]. This is in contrast to other forms of meditation, such as the focussed attention techniques (e.g. Tibetan Buddhism, Qigong) and open monitoring techniques (e.g. mindfulness, Sahaja yoga), which are generally more contemplative/concentrative in nature, effortful, and focussed on the present moment [12].

Evidence from several randomised controlled trials (RCTs) has already shown TM to be effective at improving a number of psycho-behavioural outcomes, including psychological stress, distress, anxiety, depression, quality of life, emotional and social wellbeing, and mental health [13–16]. However, there are no known studies that have examined the effects of TM on any outcome in caregivers. Findings from a small US pilot study of 31 dementia caregivers suggest that another form of relaxation therapy, mindfulness meditation, may be more effective than respite in reducing caregiver stress [17]. In view of these findings, and the results of an earlier RCT showing TM to be comparable with other kinds of relaxation therapies in reducing anxiety in patients diagnosed with anxiety neurosis [18], it is plausible that TM also may be beneficial in attenuating stress and anxiety in caregivers of persons with dementia.

Given the implications of stress on caregivers, their family and the wider community, and the favourable effects of TM on stressful symptoms; while bearing in mind the absence of data on the effects of TM in caregivers, further research in this area is justified. Utilising a randomised, wait-list controlled trial design, the aims of the current study were to (1) determine the effectiveness of a TM-based training program on psychological stress, quality of life, affect and a range of key cognitive function indices in community-dwelling caregivers of dementia sufferers, and (2) assess the feasibility of conducting a full-scale trial in this area.

## Methods

### Study design

The Transcendental Meditation for caregivers of dementia sufferers trial (TRANSCENDENT) was a pilot prospective, multi-centre, randomised wait-list controlled trial (RCT) with two parallel arms. A detailed description of the study protocol is reported elsewhere [19].

### Objectives

The project was designed to address the following objectives.

#### Primary objectives

1. Establish whether TM improves health-related quality of life in community-dwelling caregivers of dementia sufferers when compared to wait-list control (WLC).
2. Ascertain whether TM reduces psychological stress in community-dwelling caregivers of dementia sufferers when compared to WLC.

#### Secondary objectives

1. Determine whether TM improves affect in community-dwelling caregivers of dementia sufferers relative to WLC.
2. Ascertain whether TM improves cognitive performance in community-dwelling caregivers of dementia sufferers when compared to WLC.
3. Determine whether TM is cost-effective in improving health-related quality of life in community-dwelling caregivers of dementia sufferers relative to WLC.
4. Establish whether TM is associated with a greater incidence and/or severity of adverse events in community-dwelling caregivers of dementia sufferers relative to WLC.
5. Ascertain the feasibility of implementing the project as a larger RCT.

### Participants

Individual caregivers were eligible to participate in TRANSCENDENT if they were non-professional, community-dwelling caregivers of a person with diagnosed dementia; had not received previous instruction on the TM technique; were able to provide written consent; were able to speak, read and understand the English language, and were available and willing to complete all follow-up assessments and intervention sessions. Participants were excluded if they had a history of any medical condition causing moderate to severe cognitive impairment; had commenced or ceased psychotropic medication within the past six weeks; had participated in a clinical trial within the past thirty days, where psychological outcomes and quality of life were outcomes of interest; had practiced some form of mind-body therapy on a regular basis (i.e. at least once a month); consulted a psychologist/psychiatrist at least once a week, and had taken a recreational drug fifteen days prior to enrolling in the study.

The estimated size of the study population was based on an expected mean difference in quality of life (i.e. AQoL-8D utility index) of 0.1 utility points between the TM and wait-list control groups. Assuming a standard deviation of 12.5 % and 10 % attrition, a sample size of 18 patients was required in each arm. A total of 36 participants would provide 80 % power for a two-way repeated measures analysis of variance (RM-ANOVA) to detect a statistically significant difference in quality of life with a two-tailed alpha level set at 0.05.

### Setting

The project was administered by the University of South Australia, and implemented through The Positive Ageing Centre (Woodside, South Australia), and the Maharishi Invincibility Centre (Parkside, South Australia).

### Recruitment

Participant recruitment commenced in April 2013 and was completed by March 2014. A range of promotional strategies, forming part of a comprehensive recruitment campaign, were employed across multiple settings. A detailed description of the TRANSCENDENT recruitment campaign, including the recruitment yield of each strategy, will be reported in a separate publication. In brief, the strategies included the distribution of study flyers across multiple settings (i.e. University of South Australia, Adelaide Hills Council, general practice surgeries, South Australian caregiver respite agencies); the posting of regular notices about the trial on Twitter, Facebook and pertinent organisation websites; the implementation of a four-week Google Adwords campaign; the publication of study information in a state-wide periodical for senior citizens (i.e. The Senior), a national online resource on residential aged care (i.e. the DPS guide) and four association newsletters/reports; a newspaper display advertisement; the presentation of short seminars on stress management for caregivers attending three large South Australian caregiver respite agencies, and the broadcasting of three local radio station interviews, a state television news story and five newspaper stories.

Caregivers expressing interest to participate in the project were asked to contact the research assistant (by phone) for further information and screening. Those eligible to participate in the study were subsequently sent a copy of the participant information sheet and consent form to read and discuss with family or friends in order to make an informed decision about their involvement. One-week after dispatching the study information, participants were contacted by phone and their ‘capacity to give consent’ (i.e. the ability to provide a brief description of the study purpose, and outline what their involvement in the study entailed) was gauged. Participants who were able to provide a satisfactory response to these questions were invited to sign the written consent form and return it to the research assistant using the attached reply-paid envelope.

### Randomisation

Enrolled participants were randomly assigned to Transcendental Meditation® or wait-list control at a ratio of 1:1. To approximate equality of sample sizes in each study group, block randomisation was performed with computer-generated randomly permuted blocks of four by a researcher not involved in the treatment assignment process. Randomisation codes were held in sequentially numbered opaque sealed envelopes and each were selected by the research assistant (who was unaware of the allocation sequence) in consecutive order at the time of participant enrolment.

### Outcomes

The primary outcomes of TRANSCENDENT were Health-related quality of life (HR-QoL) and stress. HR-QoL index scores were measured using the paper-based, self-administered, 35-item Assessment of Quality of Life 8-dimension (AQoL-8D) instrument. Stress was measured using the WebNeuro test battery; this internet-based, self-administered, neurocognitive assessment tool has demonstrated excellent convergent validity in healthy young to middle-aged adults [20], and has been used in a number of studies looking at changes in cognitive function in obese [21] and diabetic populations [22], and children with disruptive behaviours [23].

The WebNeuro test battery was also employed to measure the secondary outcomes of affect (i.e. emotional resilience, feelings of depression and feelings of anxiety) and cognitive performance (including psychomotor response speed, impulsivity, attention and concentration, information processing efficiency, working memory and executive function). Adverse events (including description, severity and duration of symptom, probability of symptom being related to study intervention, and action taken) were measured using a standardised adverse event record, which was self-administered by participants throughout the intervention phase of the study. Cost-effectiveness was determined using cost-utility analysis, which examined the cost and effectiveness of each intervention using the quality-adjusted life year (QALY) as its unit of effectiveness (using the AQoL-8D to derive quality of life utility scores). If a statistically significant change in quality of life was detected between groups, then the cost of each intervention (as derived from trial cost data) was combined with QALYs to estimate cost per QALY gain. The final secondary outcome, feasibility, served to evaluate the ease and practicality of reproducing the study on a larger scale; the feasibility of the trial will be reported in a separate publication. Excluding feasibility and cost-effectiveness, all outcomes were assessed at baseline (week 0), post-intervention (week 12) and follow-up (week 24), and all assessments preceded intervention exposure.

### Interventions

Participants were randomly assigned to one of two groups: (a) a 12-week (14-hour) TM training program plus 12-week follow-up, delivered face-to-face by an experienced TM instructor, or (b) a 24-week wait-list control. A detailed description of the intervention and control is reported elsewhere [19].

### Statistical analysis

Data from all completed outcome measures were entered into SPSS (v.21), and analysed by intention-to-treat. Measures of central tendency and variability were used for descriptive data where values were normally distributed. Medians and the interquartile range were used to describe data that was not normally distributed. For categorical variables, frequency distributions and percentages were used to describe categorical data. The *t*-test for independent groups was used to examine differences between groups at baseline. Outcome differences between groups, differences over time and any differential treatment effect at different points in time were examined using RM-ANOVA.

### Ethics

Human ethics approval was granted by the Human Research Ethics Committee of the University of South Australia.

## Results

**Fig. 1:**
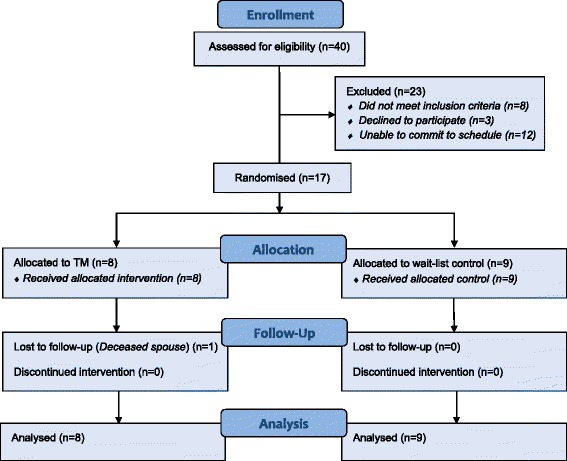
Participant flow chart

From April 2013 to March 2014, 40 individuals were screened, of which 17 were eligible for inclusion in the study (Fig. 1). The reason for exclusion in 52 % (12/23) of excepted cases was an inability to commit to the intervention schedule; this was followed by 35 % (8/23) of excluded cases not meeting the inclusion criteria. All 17 participants included in the study were randomised, with 8 assigned to TM and nine assigned to wait-list control. One participant (12.5 %) in the TM group withdrew at week 23 due to the death of a spouse; there were no withdrawals in the control group.

### Baseline demographics

The majority of participants were female (88 %), elderly (mean age: 66.12 ± 8.50 years), married to the dementia sufferer (64.7 %), provided full-time care (121.63 ± 68.04 h/week) for the person with dementia, and had been providing care for a mean duration of 5.57 ± 3.58 years. Caregivers also demonstrated a moderate degree of depression (mean WebNeuro depression score: 5.05 ± 2.10), anxiety (mean WebNeuro anxiety score: 5.12 ± 2.49), stress (mean WebNeuro stress score: 4.27 ± 1.78), and low quality of life (mean AQoL-8D utility score: 0.66 ± 0.18). None of the caregivers smoked cigarettes, and mean weekly alcohol consumption was low (4.09 ± 4.48). There were no statistically significant differences between groups at baseline for any demographic variable or outcome measure (Table 1).

**Table 1:** Baseline characteristics of participants

		TM group	WLC	*P* value
		(*n* = 8)	(*n* = 9)	
Age [years], mean (SD)		69.4 (7.3)	63.2 (8.8)	0.141
Sex, n (%)	Female	7 (87.5 %)	8 (88.9 %)	0.735
	Male	1 (12.5 %)	1 (11.1 %)	
	Total	8 (100 %)	9 (100 %)	
Number of standard alcoholic drinks consumed per week, mean (SD)		4.06 (4.63)	4.13 (4.64)	0.979
Number of cigarettes smoked per day, mean (SD)		0 (0 %)	0 (0 %)	-
Relationship to person with dementia, n (%)	Daughter/son	3 (37.5 %)	3 (33.3 %)	0.627
	Wife/Husband	5 (60.0 %)	6 (66.7 %)	
	Total	8 (100 %)	9 (100 %)	
Duration of dementia caregiving role [years], mean (SD)		6.75 (3.57)	4.21 (3.32)	0.179
Intensity of dementia caregiving role [hours/week], mean (SD)		118.06 (68.93)	125.71 (72.27)	0.837
AQoL-8D, mean (SD)	Utility score	0.65 (0.23)	0.66 (0.14)	0.877
	Independent living domain score	0.87 (0.13)	0.90 (0.11)	0.719
	Happiness domain score	0.72 (0.14)	0.76 (0.08)	0.556
	Mental health domain score	0.56 (0.12)	0.52 (0.08)	0.449
	Coping domain score	0.78 (0.14)	0.74 (0.13)	0.568
	Relationships domain score	0.69 (0.21)	0.63 (0.12)	0.498
	Self-worth domain score	0.77 (0.18)	0.84 (0.09)	0.381
	Pain domain score	0.75 (0.30)	0.76 (0.18)	0.941
	Senses domain score	0.82 (0.18)	0.91 (0.11)	0.207
WebNeuro scores, mean (SD)	Stress	4.50 (2.05)	4.06 (1.59)	0.629
	Depression	5.13 (2.33)	5.00 (2.02)	0.908
	Anxiety	5.44 (2.31)	4.83 (2.75)	0.630
	Negativity bias	2.63 (2.74)	2.11 (1.32)	0.639
	Emotional resilience	5.81 (1.81)	5.50 (2.82)	0.787
	Social skills	6.94 (1.24)	5.72 (2.49)	0.219
	Response speed	4.07 (2.54)	6.88 (2.84)	0.065
	Impulsivity	4.29 (2.69)	5.94 (2.99)	0.281
	Attention & concentration	4.50 (2.04)	3.17 (2.54)	0.264
	Information processing efficiency	3.13 (2.28)	3.89 (2.06)	0.482
	Memory	4.29 (1.72)	4.67 (1.87)	0.680
	Executive function	4.42 (3.26)	5.11 (2.52)	0.670
	Emotion identification	4.07 (2.20)	3.44 (1.21)	0.495
	Emotion bias	4.63 (2.68)	4.00 (1.99)	0.598

*AQoL-8D* assessment of quality of life (8-dimension) instrument, *SD* standard deviation, TM Transcendental Meditation®, *WLC* wait-list control.

### Quality of life

**Fig. 2:**
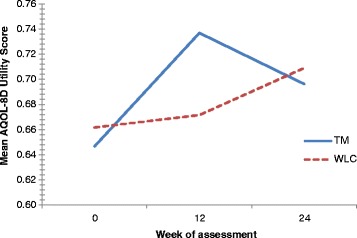
Mean Aqol-8D utility scores by treatment group at baseline, week 12 and week 24

A marginally significant increase in AQoL-8D utility scores was observed within groups over time (F (2,30) = 3.099, *p* = 0.060, η^2^ = 0.171). Whilst a trend toward greater improvement in AQoL-8D utility scores was observed in the TM group relative to the control group at week 12 (Fig. 2), differences in utility scores between groups were not statistically significant over time (F (1,15) = 0.025, *p* = 0.878, η^2^ = 0.002). Consequently, a cost-utility analysis was not performed.

Differences within groups were marginally significant for the AQOL-8D domains of mental health (F (2,30) = 3.125, *p* = 0.059, η^2^ = 0.172) and pain (F (2,30) = 3.284, *p* = 0.051, η^2^ = 0.180), and statistically significant for the physical health superdomain (F (2,30) = 3.511, *p* = 0.043, η^2^ = 0.190). There were also statistically significant differences between groups at week 12 for the domains of happiness (MD = 0.10, SD = 0.10, *p* = 0.001) and coping (MD = 0.09, SD = 0.15, *p* = 0.034), and for the mental superdomain (MD = 0.09, SD = 0.14, *p* = 0.024) (Table 2). There were no statistically significant differences between groups over time for any of the AQoL-8D domains, including independent living (F (1,15) = 0.381, *p* = 0.546, η^2^ = 0.025), happiness (F (1,15) = 0.245, *p* = 0.628, η^2^ = 0.016), mental health (F (1,15) = 0.821, *p* = 0.379, η^2^ = 0.052), coping (F (1,15) = 1.219, *p* = 0.287, η^2^ = 0.075), relationships (F (1,15) = 0.485, *p* = 0.497, η^2^ = 0.031), self-worth (F (1,15) = 0.550, *p* = 0.470, η^2^ = 0.035), pain (F (1,15) = 0.008, *p* = 0.928, η^2^ = 0.001) and senses (F (1,15) = 0.818, *p* = 0.380, η^2^ = 0.052), or for the mental health (F (1,15) = 0.897, *p* = 0.359, η^2^ = 0.056) and physical health superdomains (F (1,15) = 0.190, *p* = 0.669, η^2^ = 0.013).

**Table 2:** Changes in mean AQoL-8D scores by group at weeks 0, 12 and 24 (mean, SD)

AQoL-8D score	TM group (*n* = 8)			WLC group (*n* = 9)			Mean difference between groups at week 12^a^	Mean difference between groups at week 24^a^
	Baseline	Week 12	Week 24	Baseline	Week 12	Week 24		
Utility score	0.65 (0.23)	0.74 (0.21)	0.70 (0.21)	0.66 (0.14)	0.67 (0.10)	0.71 (0.12)	0.08 (0.19)	−0.002 (0.17)
Independent living domain score	0.87 (0.13)	0.87 (0.15)	0.88 (0.12)	0.90 (0.11)	0.91 (0.08)	0.91 (0.08)	−0.03 (0.22)	−0.01 (0.12)
Happiness domain score	0.72 (0.14)	0.81 (0.11)	0.77 (0.12)	0.76 (0.08)	0.73 (0.07)	0.74 (0.09)	0.10 (0.10)*	0.05 (0.15)
Mental health domain score	0.56 (0.12)	0.61 (0.13)	0.58 (0.12)	0.52 (0.08)	0.54 (0.05)	0.57 (0.06)	0.04 (0.12)	−0.02 (0.12)
Coping domain score	0.78 (0.14)	0.85 (0.08)	0.78 (0.13)	0.74 (0.13)	0.74 (0.12)	0.76 (0.10)	0.09 (0.15)*	−0.001 (0.17)
Relationships domain score	0.69 (0.21)	0.69 (0.24)	0.69 (0.22)	0.63 (0.12)	0.61 (0.08)	0.66 (0.11)	0.03 (0.17)	−0.02 (0.17)
Self-worth domain score	0.77 (0.18)	0.81 (0.16)	0.81 (0.15)	0.84 (0.09)	0.84 (0.08)	0.84 (0.11)	0.01 (0.16)	0.02 (0.17)
Pain domain score	0.75 (0.30)	0.84 (0.27)	0.80 (0.29)	0.76 (0.18)	0.82 (0.13)	0.84 (0.14)	0.02 (0.23)	−0.03 (0.29)
Senses domain score	0.82 (0.18)	0.90 (0.09)	0.90 (0.07)	0.91 (0.11)	0.89 (0.11)	0.92 (0.08)	0.02 (0.22)	0.006 (0.13)
Mental superdomain	0.35 (0.21)	0.42 (0.22)	0.37 (0.22)	0.30 (0.11)	0.29 (0.07)	0.33 (0.10)	0.09 (0.14)*	0.004 (0.14)
Physical superdomain	0.66 (0.24)	0.77 (0.23)	0.74 (0.21)	0.73 (0.17)	0.76 (0.15)	0.79 (0.14)	0.05 (0.27)	−0.006 (0.22)

*AQoL-8D* assessment of quality of life (8-dimension) instrument, *SD* standard deviation, *TM* Transcendental Meditation®, *WLC* wait-list control.

*Statistically significant at *p* < 0.05.

^a^Controlling for baseline values.

### Stress

**Fig. 3:**
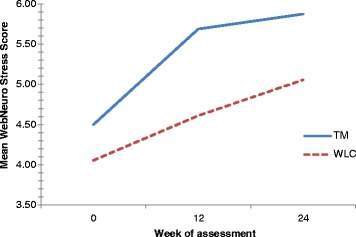
Mean WebNeuro stress scores by treatment group at baseline, week 12 and week 24

A significant improvement in WebNeuro stress scores was observed within groups over time (F (2,30) = 5.961, *p* = 0.007, η^2^ = 0.284). Changes in stress scores between groups were not statistically significant (F (1,15) = 1.350, *p* = 0.263, η^2^ = 0.083); however, there was a non-significant trend toward greater improvement in those who received TM (Fig. 3).

### Affect

**Fig. 4:**
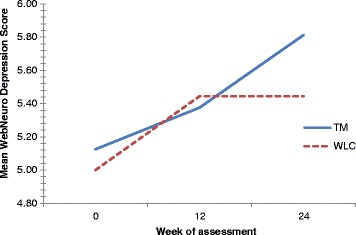
Mean WebNeuro depression scores by treatment group at baseline, week 12 and week 24

Whilst the TM group demonstrated a noticeable trend toward greater improvement in WebNeuro depression scores over time (Fig. 4), the difference in depression scores within groups (F (2,30) = 0.936, *p* = 0.403, η^2^ = 0.059) and between groups (F (1,15) = 0.034, *p* = 0.856, η^2^ = 0.002) did not reach statistical significance. Although a marginally significant improvement in anxiety scores was observed in the control group at week 12 when compared to the TM group (MD = -1.10, SD = 2.07, *p* = 0.046) (Table 3), there was no statistically significant difference within groups (F (2,30) = 1.314, *p* = 0.284, η^2^ = 0.081) or between groups (F (1,15) = <0.001, *p* = 0.998, η^2^ = <0.001) in WebNeuro anxiety scores over time.

**Table 3:** Changes in mean WebNeuro scores by group at weeks 0, 12 and 24 (mean, SD)

WebNeuro scores	TM group (*n* = 8)			WLC group (*n* = 9)			Mean difference between groups at week 12^a^	Mean difference between groups at week 24^a^
	Baseline	Week 12	Week 24	Baseline	Week 12	Week 24		
Stress	4.50 (2.05)	5.68 (1.60)	5.87 (1.98)	4.06 (1.59)	4.61 (1.22)	5.06 (1.18)	0.82 (1.93)	0.63 (2.92)
Depression	5.13 (2.33)	5.38 (2.08)	5.81 (1.81)	5.00 (2.02)	5.44 (1.36)	5.44 (1.45)	−0.13 (2.82)	0.31 (2.73)
Anxiety	5.44 (2.31)	5.44 (2.21)	5.69 (1.98)	4.83 (2.75)	6.17 (1.46)	5.56 (2.08)	−1.10 (2.07)*	−0.12 (3.16)
Negativity bias	2.63 (2.74)	3.63 (3.02)	3.81 (2.52)	2.11 (1.32)	2.17 (1.20)	2.22 (1.23)	0.97 (2.06)	1.19 (2.22)*
Emotional resilience	5.81 (1.81)	5.88 (2.00)	5.81 (2.93)	5.50 (2.82)	5.50 (2.39)	5.17 (2.14)	0.17 (3.28)	0.51 (4.82)
Social skills	6.94 (1.24)	6.75 (2.58)	6.56 (2.43)	5.72 (2.49)	5.89 (2.09)	5.11 (1.50)	−0.12 (3.64)	0.80 (3.64)
Response speed	4.07 (2.54)	3.81 (2.89)	5.00 (2.80)	6.88 (2.84)	6.63 (2.88)	6.94 (2.48)	−0.45 (5.46)	−2.26 (5.36)
Impulsivity	4.29 (2.69)	4.21 (2.86)	4.57 (2.49)	5.94 (2.99)	6.67 (2.54)	6.83 (2.37)	−1.18 (4.95)	−1.41 (4.93)
Attention & concentration	4.50 (2.04)	2.36 (1.97)	3.86 (2.14)	3.17 (2.54)	3.50 (2.86)	4.33 (2.50)	−2.18 (4.12)*	−1.28 (4.23)
Information processing efficiency	3.13 (2.28)	3.25 (2.65)	3.88 (2.08)	3.89 (2.06)	5.61 (2.32)	5.06 (2.48)	−2.01 (4.79)	−1.04 (4.79)
Memory	4.29 (1.72)	4.13 (2.83)	3.25 (2.16)	4.67 (1.87)	4.83 (2.33)	4.61 (2.73)	−1.17 (3.51)	−1.62 (4.13)
Executive function	4.42 (3.26)	5.75 (3.05)	4.38 (2.72)	5.11 (2.52)	4.94 (2.31)	5.61 (2.26)	1.51 (4.02)	−0.50 (3.73)
Emotion identification	4.07 (2.20)	4.50 (1.95)	4.94 (1.45)	3.44 (1.21)	4.61 (3.38)	4.67 (2.02)	−0.66 (4.92)	−0.06 (3.20)
Emotion bias	4.63 (2.68)	4.69 (2.32)	5.00 (1.75)	4.00 (1.99)	4.72 (3.48)	5.11 (1.41)	−0.63 (4.23)	−0.37 (2.59)

*TM* Transcendental Meditation®, *WLC* wait-list control.

*Statistically significant at *p* < 0.05.

^a^Controlling for baseline values.

### Cognitive function

#### WebNeuro response speed score

**Fig. 5:**
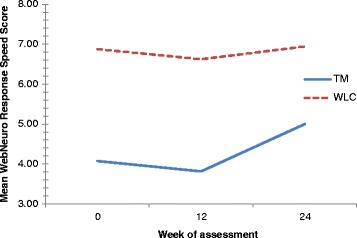
Mean WebNeuro response speed scores by treatment group at baseline, week 12 and week 24

There was no significant improvement in WebNeuro response speed scores within-groups over time (F (2,26) = 1.125, *p* = 0.340, η^2^ = 0.080). Changes in scores between groups over time were statistically significant, in favour of TM (F (1,13) = 5.774, *p* = 0.032, η^2^ = 0.308) (Fig. 5).

**Fig. 6:**
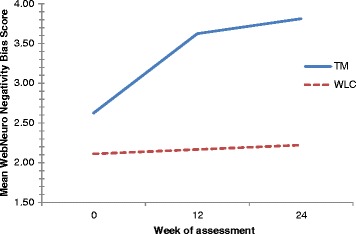
Mean WebNeuro negativity bias scores by treatment group at baseline, week 12 and week 24

A marginally significant improvement in WebNeuro negativity bias scores was observed within-groups over time (F (2,30) = 3.20, *p* = 0.055, η^2^ = 0.176). Changes in scores between groups were not statistically significant (F (1,15) = 1.499, *p* = 0.240, η^2^ = 0.091). However, a noticeable trend toward greater improvement was observed in the TM group (Fig. 6). There were no statistically significant changes between-groups over time in WebNeuro emotional resilience (F (1,15) = 0.200, *p* = 0.661, η^2^ = 0.013), social skills (F (1,15) = 1.644, *p* = 0.219, η^2^ = 0.099), impulsivity (F (1,13) = 2.771, *p* = 0.120, η^2^ = 0.176), attention and concentration (F (1,14) = 0.008, *p* = 0.930, η^2^ = 0.001), information processing (F (1,15) = 2.481, *p* = 0.136, η^2^ = 0.142), memory (F (1,14) = 1.705, *p* = 0.213, η^2^ = 0.109), executive function (F (1,13) = 0.027, *p* = 0.871, η^2^ = 0.002), emotion identification (F (1,15) = 0.090, *p* = 0.768, η^2^ = 0.006) and emotion bias scores (F (1,15) = 0.026, *p* = 0.874, η^2^ = 0.002).

### Adverse events

No participants in the control group reported an adverse event. Five participants in the TM group reported a total of eight adverse events. In 7 (88 %) cases, adverse events were short-lived (≤3 h duration). Adverse events were of moderate intensity in five (67 %) cases, mild intensity in one (13 %) case, and severe (i.e. sciatica) in one (13 %) case. In all reports, participants believed the event ‘possibly’ related to TM. Events were primarily neurological in nature, with one report each of headache, neck and shoulder pain, anger, restless feet, pins and needles, sciatica and blurred vision. No adverse events necessitated referral to a health professional, with 4 (50 %) cases requiring conservative action only (e.g. change of position, distraction, relaxation), 3 (38 %) cases requiring no action, and one case (13 %) necessitating single administration of a mild analgesic.

## Discussion

This is the first known randomised controlled trial to examine the safety and effectiveness of TM for psychological stress, quality of life, affect and cognitive performance in dementia caregivers. The findings of this trial suggest that a 12-week TM training program offers little benefit over wait-list control for most outcomes; although, there are some areas where TM shows promise.

Caregivers of dementia sufferers demonstrate poorer quality of life relative to the general population [24–26]. This study substantiates these findings, with the mean AQoL-8D (quality of life) index score for caregivers at baseline shown to be 21 % lower than AQoL-8D population norms for Australians aged ≥65 years (i.e. 0.66 vs. 0.84) [26]. Lower quality of life was evident amongst caregivers relative to population norms across all AQoL-8D domains except for independent living and senses.

Despite the poor quality of life of dementia caregivers, and the significant differences between groups in several AQoL-8D domain scores (i.e. happiness, coping, mental superdomain) at twelve weeks (in favour of TM), TM did not significantly improve AQoL-8D utility or domain scores over time when compared with wait-list control. This is not consistent with the findings of other randomised controlled trials, which have shown TM to significantly improve the quality of life of adults with breast cancer [16], congestive heart failure [27] and HIV [28]. Although these discordant findings may be attributed to the small sample size of the current study, two of the aforementioned trials were also small studies [27,28], with sample sizes of less than 24 participants. Duplication of the current study with a much larger sample size, together with the use of additional measures of quality of life (including caregiver-specific measures such as ProQolid), should further our understanding of the effects of TM on the quality of life of dementia caregivers.

The considerable level of psychological stress reported by dementia caregivers in the current study corroborates the findings of earlier reports [29,30], drawing attention to the negative effects of caring for a person with dementia [31]. The current study demonstrated that both TM and wait-list control significantly improved stress scores in dementia caregivers over time; this points toward the possible influence of contextual factors. Timeout from the caregiving role (i.e. respite) to complete the study assessments and the opportunity to speak to fellow caregivers post-assessment - both of which may alleviate caregiver burden [32]–may have contributed to improvements in stress scores in both groups. Notwithstanding, there was some indication that caregivers in the TM group experienced a greater degree of improvement in stress levels, suggesting a possible intervention effect. Whilst the positive effects of TM on stress are well-documented [14,33,34], the statistical and clinical significance of these effects needs to be substantiated by data from larger trials. Based on the findings of the current study, we calculate that a total sample size of 60 would be required to detect a mean difference in WebNeuro stress score of 0.8 between the TM and wait-list control groups (assuming a standard deviation of 1.6, 10 % attrition, a two-tailed alpha level of 0.01, and 90 % power for a two-way repeated measures analysis of variance).

The current study failed to detect a significant difference in anxiety and depression scores between the TM group and wait-list control group post-treatment. This was an unexpected finding as high-level evidence supports the effectiveness of TM for anxiety [11,35], and to a lesser extent, depression [32]. Given that there was a trend toward greater improvement in depression scores in the TM group, it is likely that the study sample was not large enough to detect a significant difference between groups. It is also conceivable that the WebNeuro test battery was not sufficiently sensitive to detect subtle changes in affect amongst caregivers [20]. Thus, the utilisation of more sensitive measures of affect should be given due consideration in future studies.

There is some indication that TM may impact positively on several aspects of cognitive function in dementia caregivers. Principally, TM significantly improved response speed scores (as measured by the motor tapping test) when compared with wait-list control. Improvements in response speed are indicative of increased psychomotor speed, attention and coordination, and may be a sign of improved affect [36]. This, together with a trend toward greater improvement in negativity bias scores (i.e. the tendency to see oneself and the world as negative), coincides with the non-significant improvement in depression scores observed in participants receiving TM. Given the number of indicators pointing toward an improved affect in dementia caregivers exposed to TM, there is clear justification to explore this relationship further.

A number of reviews of TM have drawn attention to the absence or poor reporting of adverse events in TM trials to date [35,37]. Accordingly, this is possibly the only known trial to report in sufficient detail the safety of TM. Adverse events reported by participants exposed to TM were generally transient, easily managed and of mild to moderate intensity; the events were also only weakly related to the intervention. The nature of the symptoms, which were primarily neurological, suggest that the effects were probably attributed to prolonged sitting rather than to TM per se, with prolonged sitting known to exacerbate sciatica, paraesthesia and arthralgia [38]; further, a change of position alleviated these symptoms in some cases. Another explanation is that TM increased participant self-awareness, resulting in greater attention given to pre-existing conditions; although, this has yet to be substantiated. Improved reporting of adverse events in future trials of TM will provide additional insight into the safety of this therapy.

There are some limits to the conclusions that can be drawn from this study. Firstly, despite the implementation of a comprehensive 12-month recruitment campaign, the required sample size could not be reached, and as a result, the study was underpowered; hence, it is likely that for most study outcomes, the absence of any significant difference between groups may have been the product of a type 2 error (i.e. false negative result) [39,40]. Similarly, where statistically significant differences were detected between groups, the possibility of a type 1 error (i.e. false positive result) cannot be excluded [39,40]. These limitations must be considered alongside the strengths of the study, including the robust randomised controlled trial design, the use of valid and reliable outcome measures, the homogeneity of the sample, the high-retention rate, and the medium-term (i.e. six-month) duration of the trial.

Given the limitations of the study, it would be premature to propose any recommendations for clinical practice. However, the experiences gained throughout this pilot study make it possible to put forward several recommendations for future research. First, it is evident that the study protocol is feasible to conduct as a larger clinical trial, noting that there will need to be some minor revisions; namely, the provision of respite for dementia sufferers, assistance with transportation to and from the study site, and greater flexibility with the scheduling of appointments. Second, the inclusion of caregiver-specific measures of quality of life and more sensitive measures of depression and anxiety should be given due consideration; although, the burden of these additional assessments should be weighed against the possible impact on participant recruitment and retention.

## Conclusions

The findings of this pilot study lend little support to the use of TM for the improvement of psychological stress, affect and cognitive performance in dementia caregivers. However, the study does draw attention to a number of areas where TM shows promise in this population; specifically, psychological stress, depression, negativity bias, response speed and several quality of life domains. Replication studies, which take into account the recommendations put forward by the authors, will enable firmer conclusions to be drawn about the effectiveness of TM in the dementia caregiver population.
